# Alcohol Consumption and Incident Cataract Surgery in Two Large UK Cohorts

**DOI:** 10.1016/j.ophtha.2021.02.007

**Published:** 2021-06

**Authors:** Sharon Y.L. Chua, Robert N. Luben, Shabina Hayat, David C. Broadway, Kay-Tee Khaw, Alasdair Warwick, Abigail Britten, Alexander C. Day, Nicholas Strouthidis, Praveen J. Patel, Peng T. Khaw, Paul J. Foster, Anthony P. Khawaja, UK Biobank Eye and Vision Consortium

**Affiliations:** 1 NIHR Biomedical Research Centre at Moorfields Eye Hospital NHS Foundation Trust & UCL Institute of Ophthalmology, London, United Kingdom; 2 Department of Public Health and Primary Care, University of Cambridge, Cambridge, United Kingdom; 3 Department of Ophthalmology, Norfolk & Norwich University Hospital, Norwich, United Kingdom; 4 UCL Institute of Cardiovascular Science, London, United Kingdom; 5 MRC Epidemiology Unit, University of Cambridge, Cambridge, United Kingdom

**Keywords:** Alcohol, Cataract, Cataract surgery, EPIC-Norfolk, Longitudinal observational cohort, UK Biobank, Wine, BMES, Blue Mountains Eye Study, BMI, body mass index, CI, confidence interval, EPIC, European Prospective Investigation of Cancer, HR, hazard ratio, LDL-C, low-density lipoprotein cholesterol, SD, standard deviation

## Abstract

**Purpose:**

To examine the association of alcohol consumption and type of alcoholic beverage with incident cataract surgery in 2 large cohorts.

**Design:**

Longitudinal, observational study.

**Participants:**

We included 469 387 participants of UK Biobank with a mean age of 56 years and 23 162 participants of European Prospective Investigation of Cancer (EPIC)-Norfolk with a mean age of 59 years.

**Methods:**

Self-reported alcohol consumption at baseline was ascertained by a touchscreen questionnaire in UK Biobank and a food-frequency questionnaire in EPIC-Norfolk. Cases were defined as participants undergoing cataract surgery in either eye as ascertained via data linkage to National Health Service procedure statistics. We excluded participants with cataract surgery up to 1 year after the baseline assessment visit or those with self-reported cataract at baseline. Cox proportional hazards models were used to examine the associations of alcohol consumption with incident cataract surgery, adjusted for age, sex, ethnicity, Townsend deprivation index, body mass index (BMI), smoking, and diabetes status.

**Main Outcome Measures:**

Incident cataract surgery.

**Results:**

There were 19 011 (mean cohort follow-up of 95 months) and 4573 (mean cohort follow-up of 193 months) incident cases of cataract surgery in UK Biobank and EPIC-Norfolk, respectively. Compared with nondrinkers, drinkers were less likely to undergo cataract surgery in UK Biobank (hazard ratio [HR], 0.89; 95% confidence interval [CI], 0.85–0.93) and EPIC-Norfolk (HR, 0.90; 95% CI, 0.84–0.97) after adjusting for covariables. Among alcohol consumers, greater alcohol consumption was associated with a reduced risk of undergoing cataract surgery in EPIC-Norfolk (*P* < 0.001), whereas a U-shaped association was observed in the UK Biobank. Compared with nondrinkers, subgroup analysis by type of alcohol beverage showed the strongest protective association with wine consumption; the risk of incident cataract surgery was 23% and 14% lower among those in the highest category of wine consumption in EPIC-Norfolk and UK Biobank, respectively.

**Conclusions:**

Our findings suggest a lower risk of undergoing cataract surgery with low to moderate alcohol consumption. The association was particularly apparent with wine consumption. We cannot exclude the possibility of residual confounding, and further studies are required to determine whether this association is causal in nature.

Age-related cataract is the leading cause of visual impairment worldwide and is a significant public health burden.^1^ According to the Global Burden of Disease, Injuries and Risk Factors Study, cataract accounted for 35% of blindness and 25% of visual impairment in adults aged 50 years and older in 2015.^1^ With an aging population and greater life expectancy, the number of people with cataract is expected to increase.^2^ Currently, the only available treatment for cataract is surgical extraction of the lens. Thus, identifying modifiable risk factors could help to ease the burden. Additionally, understanding risk factors for cataract can shed light on its etiology, which may in turn lead to new treatment strategies. Alcohol consumption is associated with a wide range of chronic diseases, including cardiovascular diseases, diabetes mellitus, and cancers.^3^, ^4^, ^5^ The observed relationship is often nonlinear, with low to moderate alcohol consumption being protective and higher consumption harmful.^6^^,^^7^

Studies reporting the association between alcohol consumption and cataract have been inconsistent.^8^, ^9^, ^10^, ^11^, ^12^ Heavy drinking^8^^,^^9^^,^^11^^,^^13^ or hard liquor consumption^12^ has been associated with increased risk of cataract or cataract surgery. However, moderate alcohol consumption or wine consumption has been associated with less cataract or cataract surgery,^11^^,^^13^^,^^14^ and other studies have found no relationship.^15^^,^^16^ Evidence from prospective studies remains limited, and these have shown inconsistent findings.^8^^,^^9^^,^^15^ The Blue Mountains Eye Study (BMES) reported moderate alcohol consumption was associated with reduced likelihood of cataract surgery,^9^ whereas increased risk of cataract surgery was reported among Swedish women with daily consumption of ≥1 alcoholic drinks.^8^ In contrast, the Nurses Health Study reported no association of cataract surgery with alcohol intake.^15^

In this large longitudinal observational study, we examined the association between alcohol consumption and the incidence of cataract surgery in 2 independent cohort studies, the UK Biobank and European Prospective Investigation of Cancer (EPIC)-Norfolk. We further examined the dose–response relationship between alcohol consumption and cataract surgery and examined associations with subtypes of alcoholic beverage.

## Methods

### EPIC-Norfolk

#### Study Population

The EPIC is a 10-country collaborative study that started in 1989.^17^ The EPIC-Norfolk, one of the UK centers, recruited 25 639 UK residents in East Anglia aged 40 to 79 years between 1993 and 1997.^18^ The study was approved by the Norwich Local Research Ethics Committee. Baseline examination comprised a clinic visit to obtain anthropometric measurements and completion of a detailed questionnaire to assess demographic, health, and lifestyle information. Choices for ethnicity included White, Black, Indian, Pakistan, Bangladesh, Chinese, and others. Townsend deprivation index was determined according to the participants’ postcode at recruitment and the corresponding output area from the preceding national census. The index was calculated on the basis of the output area’s employment status, home and car ownership, and household condition; the higher and more positive the index, the more deprived an area. Smoking status was defined as self-reported history of smoking cigarettes in the past or those who were currently smoking at baseline. Diabetes status was determined by self-report at baseline. Participants completed a questionnaire that evaluated their occupational and leisure physical activity. Physical activity at work was classified as 4 categories: sedentary, standing, physical work, and heavy manual work. Leisure activity assessed the time spent cycling, attending keep fit classes, swimming, or jogging in winter and summer.^19^ Height was measured using a stadiometer (Chasemores), and weight was measured using digital scales (Salter, Tonbridge). Body mass index (BMI) was calculated as weight (kilograms)/height (meters squared).

#### Assessment of Alcohol Consumption

In EPIC-Norfolk, baseline usual alcohol intake was ascertained by a validated food frequency questionnaire.^20^^,^^21^ The food frequency questionnaire measures a participant’s usual food and drink intake during the previous year and contains a list of 130 items. Participants were asked to indicate their usual consumption, choosing from 9 frequency categories, which ranged from “never or less than once per month” to “6 or more times per day” (Table S1, available at www.aaojournal.org). One unit of alcohol is equivalent to 1 glass of wine; half a pint of beer, lager, or cider; or 1 single measure of spirits. Alcohol intake in grams was calculated using a custom-designed dietary assessment software program (Compositional Analyses from Frequency Estimates).^22^ We also categorized the intake of specific alcoholic beverages (wine, beer, and spirits) into tertiles based on the absolute alcohol intake from each beverage.

### UK Biobank

#### Study Population

UK Biobank is a large community-based cohort of 502 504 UK residents registered with the National Health Service and aged 40 to 69 years at enrollment. Baseline examinations were carried out between 2006 and 2010 at 22 study assessment centers. The North West Multi-center Research Ethics Committee approved the study in accordance with the principles of the Declaration of Helsinki. The overall study protocol (http://www.ukbiobank.ac.uk/resources/) and protocols for individual tests (http://biobank.ctsu.ox.ac.uk/crystal/docs.cgi) are available online. All participants provided informed consent. Participants answered a detailed questionnaire that covers a wide range of demographic, health, and lifestyle information.^23^ The choices for ethnicity included White (English/Irish or other White background), Asian or British Asian (Indian/Pakistani/Bangladeshi or other Asian background), Black or Black British (Caribbean, African, or other Black background), Chinese, mixed (White and Black Caribbean or African, White, and Asian, or other mixed background), or other ethnic group (not defined). Townsend deprivation index was determined according to the participants’ postcodes using the same method as detailed earlier for EPIC-Norfolk. Smoking status was determined by self-report. Diabetes status was defined by self-report of diabetes mellitus or use of diabetes medications. Physical activity was assessed using the short-form International Physical Activity Questionnaire,^24^ which examined the frequency and duration of walking, moderate-intensity activity, and vigorous-intensity activity.^25^ Weight was measured with the BV-418 MA body composition analyzer (Tanita). Height was measured using a Seca 202 height measure.

#### Assessment of Alcohol Consumption

Information on baseline alcohol consumption was obtained from a touchscreen self-administered questionnaire in UK Biobank. Although the questionnaire has not been formally validated, multiple previous studies have demonstrated expected associations with alcohol.^26^^,^^27^ Participants were asked to indicate their usual consumption, choosing from 6 frequency categories, which were “never,” “special occasions only,” “1–3 times a month,” “1–2 times a week,” “3–4 times a week,” and “daily or almost daily.” Alcohol frequency was classified into 4 groups (≤1–3 times a month, 1–2 times a week, 3–4 times a week, and daily or almost daily) among drinkers (Table S1, available at www.aaojournal.org). We further assessed the consumption of different types of alcohol (red wine; white wine and champagne; beer and cider; spirits) among drinkers who reported alcohol consumption at least 1 to 2 times per week. Drinking frequency for each type of alcohol was categorized into 1 of 3 groups (1 to 2, 3 to 4, and ≥5 drinks per week).

### Ascertainment of Incident Cataract Surgery in EPIC-Norfolk and UK Biobank

Incident cataract surgery was ascertained via linkage to hospital procedure records, namely, Hospital Episode Statistics for England, Scottish Morbidity Record for Scotland, and Patient Episode Database for Wales. It was defined as cataract surgery in either eye, and the date of event was defined as the date of first eye cataract surgery in participants undergoing bilateral sequential surgery. Participants were determined to have had cataract surgery if they had an OPCS Classification of Interventions and Procedures 4 code of C71.2 - "Phacoemulsification of lens" or C75.1 - "Insertion of prosthetic replacement for lens." We excluded participants with cataract surgery up to 1 year after the baseline assessment visit because this may indicate visually significant cataract having been present at baseline. Participants with self-reported cataract at baseline were also excluded from this study. The reliability of self-reported cataract has been evaluated in the Physicians’ Health Study.^28^ Self-reported cataract was shown to be a good indicator of lens opacification compared with medical record data.

### Definition of Covariables in EPIC-Norfolk and UK Biobank

Demographic characteristics in the analysis included age at baseline, sex, ethnicity (White or non-White), and Townsend deprivation index. Health and lifestyle factors included BMI, smoking status (never smoked vs. ever smoked), diabetes status (yes vs. no), and physical activity. Physical activity was categorized as low, moderate, and high in UK Biobank,^25^ and EPIC-Norfolk participants were classified as inactive, moderately inactive, moderately active, or active.^29^

### Statistical Analysis

The baseline characteristics of EPIC-Norfolk and UK Biobank participants are presented as means (standard deviation [SD]) for continuous variables and numbers (percentage) for categorical variables. We conducted a survival analysis, and participants were censored at the following end points: date of first cataract surgery, date of death, or end of the data linkage (March 31, 2015, for EPIC-Norfolk and March 31, 2017, for UK Biobank), whichever came first. The data linkage to identify incident cataract surgery was done on a national level and would therefore even capture participants who had moved within the country. However, if participants had moved abroad, or if they have opted out of national statistics collection, then we would miss if they had cataract surgery. The numbers of such participants are likely to be low. Cox proportional hazards models were used to examine associations with incident cataract surgery. Given that nondrinkers may differ from current drinkers in aspects other than just alcohol consumption (e.g., people may decrease their alcohol consumption as they age or become ill),^30^ we carried out a 2-step analysis. The first step was to compare the risk of incident cataract surgery in alcohol drinkers with that of nondrinkers. The second step was to examine for a dose response for the association between alcohol consumption and incident cataract surgery among drinkers only; we compared across quartiles of absolute alcohol intake in EPIC-Norfolk and across the frequency of alcohol consumption in UK Biobank. We further assessed the risk of incident cataract surgery with consumption of different types of alcoholic beverage. All associations were examined using univariable and multivariable models. Multivariable models were adjusted for age, sex, ethnicity, Townsend deprivation index, BMI, smoking, and diabetes status. In a sensitivity analysis, physical activity was also adjusted for in the multivariable models because of its association with alcohol intake^31^ and cataract risk.^32^ Physical activity was not included in the primary analysis given the significant number of participants with missing data in UK Biobank. We additionally examined the association between alcohol intake and incident cataract surgery without excluding those with self-reported cataract. We constructed correlation and variance-covariance matrices for the continuous explanatory variables we examined (age, Townsend deprivation index, and BMI); there was no evidence for multicollinearity. Data analysis was performed using STATA software (version 16, StataCorp LP).

## Results

**Figure 1:**
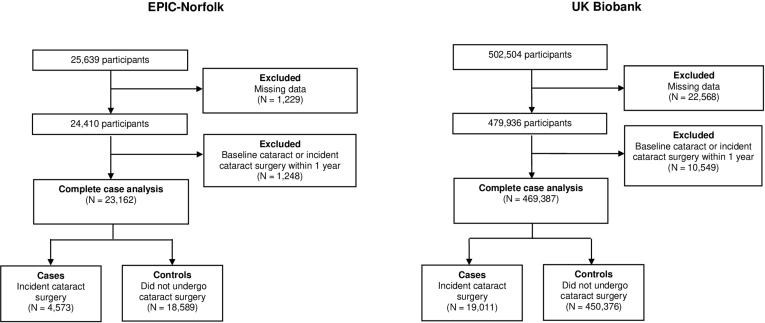
Flowchart of participants included in the European Prospective Investigation of Cancer (EPIC)-Norfolk and UK Biobank cohorts.

Of the 25 639 EPIC-Norfolk participants, a total of 23 162 participants were included in this analysis after excluding 1229 with missing data and 1248 with baseline cataract or incident cataract surgery within 1 year (Fig 1). The mean follow-up time was 193 months (SD, 62 months), during which time 4573 participants underwent cataract surgery. Of the 502 504 UK Biobank participants, 469 387 participants were included after the exclusion of 22 568 participants with missing data and 10 549 participants with baseline cataract or incident cataract surgery within 1 year. The mean follow-up time was 95 months (SD, 15 months), during which time 19 011 participants underwent cataract surgery. In both cohorts, compared with participants who were included, those excluded were older, more likely women (only in EPIC-Norfolk) and non-White (only in UK Biobank), more likely to reside in a more deprived area, more likely to have a higher BMI, more likely to have ever smoked, more likely to have diabetes, and less likely to be drinkers (all *P <* 0.001) (Tables S2 and S3, available at www.aaojournal.org). The length of follow-up was considerably longer in the EPIC-Norfolk study than the UK Biobank. This does not alter the interpretation of the hazard ratio (HR) for either study; the HR reflects the ratio of the instantaneous risk at any point in time and therefore applies at any period of follow-up in either study. Notably, we confirmed that the proportional hazards assumption was met in both EPIC-Norfolk and UK Biobank.

Table 1 presents the baseline characteristics of both EPIC-Norfolk and UK Biobank participants included in the study. Compared with UK Biobank participants, EPIC-Norfolk participants were slightly older and more likely to be White, to live in a less-deprived area, to have a lower BMI, or to have ever smoked. The duration of follow-up was twice as long in EPIC-Norfolk compared with UK Biobank (193 months vs. 95 months). A greater proportion of participants were alcohol drinkers at baseline in UK Biobank compared with EPIC-Norfolk (92% vs. 81%). Among the drinkers in UK Biobank, 67%, 55%, 53%, and 37% consumed red wine, white wine/champagne, beer/cider, and spirits, respectively. Among the drinkers in EPIC-Norfolk, 85%, 57%, and 53% consumed wine, beer, and spirits, respectively.

**Table 1:** Comparison of Baseline Characteristics between EPIC-Norfolk and UK Biobank Participants

	EPIC-Norfolk	UK Biobank	*P* Value
Recruitment yrs	1993–1999	2006–2010	
Sample size	23 162	469 387	
Age (yrs), mean (SD)	58.8 (9.2)	56.3 (8.1)	<0.001
Sex, n (%)			0.83
Men	10 575 (45.7)	214 046 (45.6)	
Women	12 587 (54.3)	255 341 (54.4)	
Ethnicity, n (%)			<0.001
White	23 083 (99.7)	445 610 (94.9)	
Non-White	79 (0.3)	23 777 (5.1)	
Townsend deprivation index, mean (SD)	-2.1 (2.1)	-1.3 (3.1)	<0.001
BMI (kg/m^2^), mean (SD)	26.3 (3.9)	27.4 (4.8)	<0.001
Smoking status, n (%)			<0.001
Never smoked	10 713 (46.3)	258 118 (55.0)	
Ever smoked	12 449 (53.7)	211 269 (45.0)	
Diabetes status, n (%)			0.022
No	22 473 (97.0)	446 241 (95.1)	
Yes	689 (3.0)	23 146 (4.9)	
Alcohol status at baseline, n (%)			<0.001
Nondrinker or former drinker	4516 (19.5)	37 127 (7.9)	
Current drinker	18 646 (80.5)	432 260 (92.1)	
Incident cataract surgery, n (%)			<0.001
No	18 589 (80.3)	450 376 (95.9)	
Yes	4573 (19.7)	19 011 (4.1)	
Duration of follow-up (mos), mean (SD)	193 (62)	95 (15)	<0.001

BMI = body mass index; EPIC = European Prospective Investigation of Cancer; N = sample size; SD = standard deviation.

### Step 1: Comparing Alcohol Drinkers with Nondrinkers

**Figure 2:**
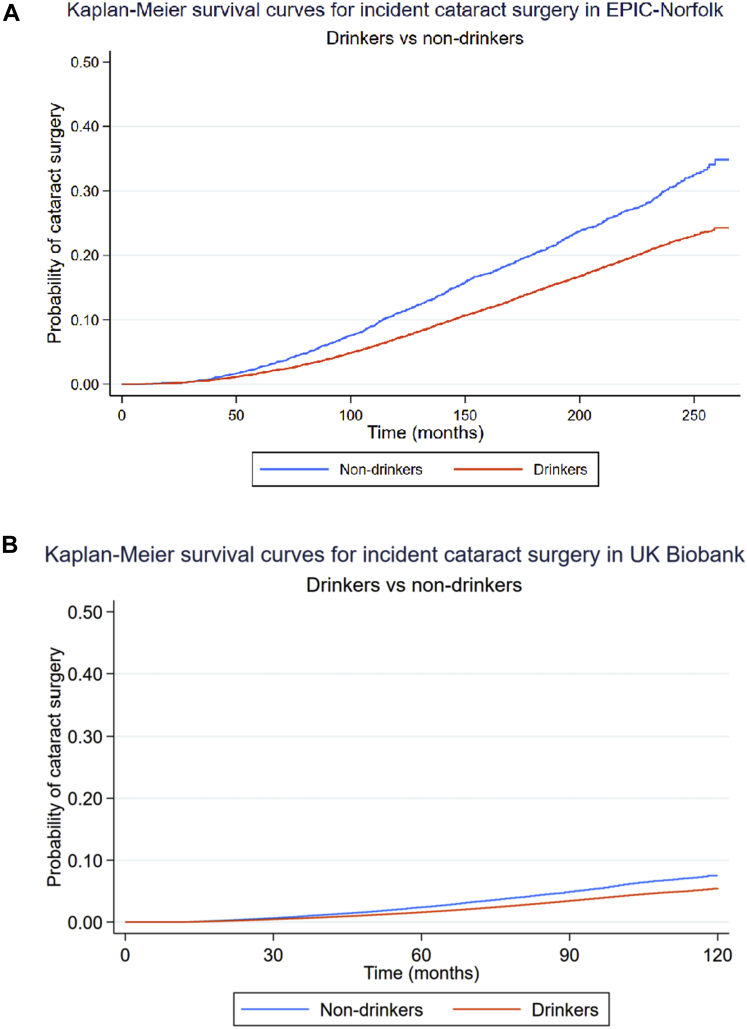
Kaplan–Meier survival curves for incident cataract surgery among drinkers and nondrinkers in (A) EPIC-Norfolk and (B) UK Biobank cohorts. EPIC = European Prospective Investigation of Cancer.

In unadjusted analyses, alcohol drinkers were less likely to undergo cataract surgery than nondrinkers in both EPIC-Norfolk (HR, 0.68; 95% confidence interval [CI], 0.64–0.73; *P <* 0.001) and UK Biobank (HR, 0.70; 95% CI, 0.67–0.73; *P <* 0.001). Figure 2 shows the unadjusted survival functions for incident cataract surgery among drinkers compared with nondrinkers in EPIC-Norfolk and UK Biobank. After adjusting for covariables, the associations remained statistically significant; compared with nondrinkers, drinkers were less likely to undergo cataract surgery in EPIC-Norfolk (HR, 0.90; 95% CI, 0.84–0.97; *P* = 0.004) and UK Biobank (HR, 0.89; 95% CI, 0.85–0.93; *P <* 0.001).

### Step 2: Examining for a Dose–Response Association among Alcohol Drinkers Only

The dose–response associations between alcohol consumption and incident cataract surgery among alcohol drinkers only are shown in Table 2. In EPIC-Norfolk, the risk of incident cataract surgery was progressively lower with greater alcohol consumption (*P <* 0.001). In the multivariable adjusted analysis, participants in the third and highest quartiles of alcohol intake had 14% and 18% lower risk of incident cataract surgery, respectively, compared with those in the lowest quartile of alcohol intake. In UK Biobank, there was a U-shaped association between alcohol consumption and cataract surgery (Table 2). Compared with participants who drank 1 to 3 times or less per month, those who drank 1 to 2 times and 3 to 4 times per week had 7% and 6% lower risk of incident cataract surgery, respectively, whereas no significant association was observed among those with daily or almost daily alcohol consumption. Compared with participants who consumed alcohol 1 to 2 times per week or 3 to 4 times per week, those who drank daily or almost daily had 6% (95% CI, 1.02–1.12, *P* = 0.010) and 5% (95% CI, 1.00–1.10, *P* = 0.05) higher risk of incident cataract surgery, respectively. This demonstrates a significant increase in cataract surgery risk with the highest frequency of intake compared with moderate frequency, supporting a U-shaped association.

**Table 2:** Multivariable Dose–Response Associations of Alcohol Consumption with Incident Cataract Surgery among Alcohol Drinkers in EPIC-Norfolk and UK Biobank

	No. of Incident Cases	No. at Risk	Univariable Model		Multivariable Model	
			Hazard Ratio (95% CI)	*P* Value	Hazard Ratio (95% CI)	*P* Value
Alcohol consumers in EPIC-Norfolk (n = 18 646)						
By quartiles of total weekly alcohol intake						
Lowest intake (≤14.10 g/≤1.76 units)	998	4590	Ref		Ref	
Quartile 2 (14.23–43.70 g/1.77–5.46 units)	899	4654	0.88 (0.80–0.96)	**0.005**	0.92 (0.84–1.01)	0.07
Quartile 3 (43.83–88.53 g/5.47–11.07 units)	826	4645	0.80 (0.73–0.88)	**<0.001**	0.86 (0.79–0.95)	**0.002**
Highest intake (≥88.78 g/≥11.09 units)	732	4757	0.70 (0.64–0.77)	**<0.001**	0.82 (0.74–0.90)	**<0.001**
*P* for trend				**<0.001**		**<0.001**
Alcohol consumers in UK Biobank (n = 432 260)						
By frequency of alcohol consumption						
1–3 times or less per month	4646	107 112	Ref		Ref	
1–2 times per week	4380	121 159	0.82 (0.79–0.86)	**<0.001**	0.93 (0.89–0.97)	**0.001**
3–4 times per week	3887	108 897	0.81 (0.78–0.85)	**<0.001**	0.94 (0.90–0.98)	**0.005**
Daily or almost daily	4064	95 092	0.98 (0.94–1.03)	0.42	0.97 (0.93–1.01)	0.16
*P* for trend				0.18		0.22

BMI = body mass index; CI = confidence interval; EPIC = European Prospective Investigation of Cancer.

Multivariable models were adjusted for age, sex, ethnicity, Townsend deprivation index, BMI, smoking, and diabetes status.

Bold values denote statistical significance at the *P <* 0.05 level.

Alcohol consumption was quantified by absolute intake in EPIC-Norfolk (presented in both grams and units per week) and by frequency of intake in UK Biobank.

One unit of alcohol (8 g) is equivalent to 1 glass of wine; half a pint of beer, lager, or cider; or 1 single measure of spirits.

### Analysis of Alcoholic Beverage Subtypes

We then examined the association of consumption of different alcoholic beverage types with incident cataract surgery. In EPIC-Norfolk, wine consumption was most strongly associated with a reduced risk of cataract surgery (Table 3). The risk for incident cataract surgery decreased in a dose–response manner with increasing wine consumption (*P <* 0.001). Compared with nondrinkers, wine consumption in the second tertile and third tertile had 19% and 23% lower risk of incident cataract, respectively. Beer consumption in the second tertile and spirits consumption in the third tertile showed a 13% and 14% lower risk of incident cataract surgery, respectively. Table 4 shows the association between different types of alcohol and incident cataract surgery in UK Biobank. Compared with nondrinkers, the risk of cataract surgery was 14% lower among red wine consumers, regardless of amount of consumption. Likewise, compared with nondrinkers, white wine/champagne consumers had at least 10% lower risk of incident cataract surgery regardless of amount of consumption. In contrast, although moderate consumers of beer and cider or spirits had a lower risk of incident cataract surgery compared with nondrinkers, the most frequent consumers did not have a significantly different risk.

**Table 3:** Hazard Ratio of Incident Cataract Surgery Across Different Alcohol Beverages among Drinkers Compared with Nondrinkers in EPIC-Norfolk

Amount of Alcohol Intake (g)	No. of Incident Cases	No. at Risk	Univariable Model		Multivariable Model	
			Hazard Ratio (95% CI)	*P* Value	Hazard Ratio (95% CI)	*P* Value
Nondrinkers	1118	4516	Ref		Ref	
Wine drinkers						
First tertile	1626	7902	0.76 (0.70–0.82)	**<0.001**	0.98 (0.91–1.06)	0.57
Second tertile	688	4169	0.58 (0.53–0.64)	**<0.001**	0.81 (0.74–0.89)	**<0.001**
Third tertile	547	3721	0.52 (0.47–0.58)	**<0.001**	0.77 (0.69–0.85)	**<0.001**
*P* for trend				**<0.001**		**<0.001**
Nondrinkers	1118	4516	Ref		Ref	
Beer drinkers						
First tertile	985	5532	0.65 (0.59–0.71)	**<0.001**	0.92 (0.84–1.01)	0.07
Second tertile	392	2524	0.56 (0.50–0.63)	**<0.001**	0.87 (0.77–0.99)	**0.033**
Third tertile	360	2567	0.52 (0.46–0.59)	**<0.001**	0.91 (0.80–1.04)	0.18
*P* for trend				**<0.001**		0.07
Nondrinkers	1118	4516	Ref		Ref	
Spirits drinkers						
First tertile	642	3591	0.64 (0.58–0.71)	**<0.001**	0.94 (0.85–1.04)	0.23
Second tertile	815	4524	0.66 (0.61–0.72)	**<0.001**	0.91 (0.83–1.00)	0.06
Third tertile	356	1842	0.76 (0.68–0.86)	**<0.001**	0.86 (0.76–0.97)	**0.016**
*P* for trend				**<0.001**		**0.009**

BMI = body mass index; CI = confidence interval; EPIC = European Prospective Investigation of Cancer.

Multivariable model adjusted for age, sex, ethnicity, Townsend deprivation index, BMI, smoking, and diabetes status.

Bold values denote statistical significance at the *P <* 0.05 level.

**Table 4:** Hazard Ratio of Incident Cataract Surgery Across the Different Alcohol Beverages among Drinkers Compared with Nondrinkers in the UK Biobank

No. of Drinks per Week	No. of Incident Cases	No. at Risk	Univariable Model		Multivariable Model	
			Hazard Ratio (95% CI)	*P* Value	Hazard Ratio (95% CI)	*P* Value
Nondrinkers	2034	37 127	Ref		Ref	
Red wine drinkers						
1–2 glasses/wk	2490	66 590	0.93 (0.89–0.98)	**0.006**	0.86 (0.81–0.91)	**<0.001**
3–4 glasses/wk	1922	51 608	0.92 (0.87–0.97)	**0.003**	0.86 (0.80–0.92)	**<0.001**
≥5 glasses/wk	3566	98 144	0.90 (0.86–0.94)	**<0.001**	0.86 (0.81–0.91)	**<0.001**
*P* for trend				**<0.001**		**<0.001**
Nondrinkers	2034	37 127	Ref		Ref	
White wine and champagne drinkers						
1–2 glasses/wk	2860	76 045	0.95 (0.91–0.99)	**0.022**	0.85 (0.80–0.91)	**<0.001**
3–4 glasses/wk	1526	42 003	0.91 (0.86–0.96)	**0.001**	0.85 (0.79–0.91)	**<0.001**
≥5 glasses/wk	2144	60 788	0.89 (0.84–0.93)	**<0.001**	0.90 (0.84–0.96)	**0.001**
*P* for trend				**<0.001**		**0.025**
Nondrinkers	2034	37 127	Ref		Ref	
Beer and cider drinkers						
1–2 pints/wk	2342	71 814	0.79 (0.76–0.83)	**<0.001**	0.84 (0.79–0.90)	**<0.001**
3–4 pints/wk	1132	33 712	0.82 (0.77–0.87)	**<0.001**	0.90 (0.83–0.98)	**0.011**
≥5 pints/wk	2532	66 810	0.92 (0.88–0.97)	**0.001**	1.03 (0.96–1.11)	0.35
*P* for trend				**<0.001**		**0.002**
Nondrinkers	2034	37 127	Ref		Ref	
Spirits drinkers						
1–2 measures/wk	2416	62 171	1.10 (1.05–1.15)	**<0.001**	0.87 (0.82–0.93)	**<0.001**
3–4 measures/wk	1080	24 573	1.24 (1.16–1.32)	**<0.001**	0.91 (0.84–0.98)	**0.011**
≥5 measures/wk	1701	35 012	1.38 (1.31–1.45)	**<0.001**	0.96 (0.89–1.03)	0.23
*P* for trend				**<0.001**		0.68

BMI = body mass index; CI = confidence interval.

Multivariable models adjusted for age, sex, ethnicity, Townsend deprivation index, BMI, smoking and diabetes status.

Bold values denote statistical significance at the *P <* 0.05 level.

### Sensitivity Analyses

After additional adjustment for physical activity, compared with nondrinkers, drinkers were less likely to undergo cataract surgery in EPIC-Norfolk (HR, 0.91, 95% CI, 0.85–0.98, *P* = 0.008) and UK Biobank (HR, 0.90, 95% CI, 0.85–0.95, *P <* 0.001). The dose–response associations of alcohol consumption and consumption of different alcoholic beverage types with incident cataract were similar (Tables S4–S6, available at www.aaojournal.org). We also performed additional analyses given the uncertain accuracy of self-reported cataract at baseline. Self-reported cataract was associated with incident cataract surgery in EPIC-Norfolk (HR, 1.42, 95% CI, 1.29–1.56, *P <* 0.001) and UK Biobank (HR, 4.97, 95% CI, 4.78–5.18, *P <* 0.001). In a sensitivity analysis without excluding participants with self-reported cataract, compared with nondrinkers, drinkers were less likely to undergo cataract surgery in EPIC-Norfolk (HR, 0.91, 95% CI, 0.85–0.97, *P* = 0.005) and UK Biobank (HR, 0.92, 95% CI, 0.87–0.97, *P* = 0.003). The dose–response associations of alcohol consumption with incident cataract were similar in both cohorts.

## Discussion

In this analysis of British adults, we report low to moderate consumption of alcohol to be associated with a reduced risk of undergoing subsequent cataract surgery; this finding was consistent between 2 independent studies with contrasting methods of ascertaining alcohol intake. The protective association was apparent whether any consumption of alcohol was compared with nonconsumption and whether the amount or frequency of alcohol intake was compared among drinkers only in dose–response analyses. We found the strongest protective association among wine drinkers.

Most previous studies examining the association between alcohol consumption and cataract surgery have been limited by their cross-sectional design.^11^, ^12^, ^13^ The Beaver Dam Eye Study reported that wine consumption was associated with less severe nuclear sclerosis and cortical opacities, whereas drinking beer was associated with increased prevalence of cortical opacities.^13^ In the BMES, compared with nondrinkers, alcohol consumption was associated with reduced prevalence of cortical cataract (OR, 0.70; 95% CI, 0.60–0.90).^11^ There have been only a small number of longitudinal studies that examined the relationship between alcohol consumption and cataract.^8^^,^^9^^,^^15^ These studies have a smaller sample size, were mainly evaluated in women, and have reported inconsistent findings. The BMES reported that moderate alcohol consumption was associated with 50% lower incidence of cataract surgery compared with abstinence or heavy alcohol consumption.^9^ In contrast, an increase of ≥1 drink per day was associated with a 7% increased risk of cataract extraction in a Swedish Mammography cohort,^8^ and the Nurses Health Study found no relationship between alcohol intake and cataract surgery in women.^15^ Furthermore, these studies did not report an association between different types of alcoholic beverage and cataract extraction, which may have been limited by the smaller sample size in each subgroup analysis. Our study is longitudinal in design and has a very large sample size. The alcohol intake dose–response analyses are different in EPIC-Norfolk (doses defined by quantity of intake) and UK Biobank (doses defined by frequency of intake) due to differences in data collection. Despite this difference, we demonstrated a dose–response relationship between alcohol intake and cataract surgery in both cohorts. Unlike previous studies, we excluded participants who had undergone cataract surgery up to 1 year from baseline to minimize the chance of reverse causality underlying our identified associations. Additionally, we evaluated the association between the amount or frequency of alcohol intake and cataract surgery among drinkers only, because nondrinkers may differ from drinkers in ways other than their alcohol consumption (e.g., unwell participants may stop drinking alcohol^30^); these dose–response analyses further support an association between alcohol intake and cataract surgery.

The findings of our study have to be taken in the context of our primary outcome, cataract surgery, which is our surrogate for visually significant cataract. Factors other than visual impairment may determine whether a person undergoes cataract surgery. Access to health care and attitudes toward surgery will have an influence. The threshold of visual impairment required to prompt a decision to undergo surgery will also vary by individual. Furthermore, given the observational nature of our study, it is not possible to determine if the protective association we observed of alcohol intake on cataract surgery is causal. The fact that the association is present whether comparing drinkers with nondrinkers, or in dose–response analyses among drinkers only, increases the chance that this association is causal. However, the association may also be due to confounding. For example, alcohol consumers may be of higher social class than nonconsumers, and it is other aspects of lifestyle and healthcare access associated with social class that is driving the association.^33^ Although we adjusted for sociodemographic factors, it is possible that these measures did not fully account for differences in social class between drinkers and nondrinkers. However, it seems unlikely that social class differences completely explain the differential cataract surgery risk across the different doses of alcohol intake; the dose–response relationship we observe supports a causal relationship. Although drinking patterns may vary by ethnicity-related cultures, this is unlikely to underlie our observed associations in EPIC-Norfolk as the participants are almost entirely White.

The majority of alcohol consumers in both EPIC-Norfolk and UK Biobank reported only low to moderate amounts of alcohol intake, which may be reflective of the healthier nature of participants in cohort studies. Therefore, we cannot make inference regarding the potential protective association of greater than moderate alcohol intake on cataract. Although we observed a dose–response association of progressively reduced cataract surgery risk with increasing alcohol intake across the low to moderate quantity or frequency range, the most frequent drinkers in UK Biobank (daily or almost daily intake) did not have a different risk of cataract surgery compared with the least frequent drinkers (Table 2). Results from the UK Biobank subset suggest a U-shaped relationship between alcohol intake and cataract surgery within the alcohol intake frequency range observed in UK Biobank; this may be analogous to the J-shaped relationship observed between alcohol intake and cardiovascular disease^7^ but truncated because of a paucity of heavy drinkers in UK Biobank. In EPIC-Norfolk, few participants reported heavy drinking (only 3.4% reported >42 units/week or >336 g/week of alcohol intake),^34^ and therefore it was not possible to examine for a U- or J-shaped relationship with sufficient statistical power. It will be a challenge for future cohort studies to ascertain the prospective effect of heavy alcohol intake, because it may be less likely for heavy drinkers to volunteer for such studies. The current guidelines for safe alcohol intake quantity are up to 14 units/week (equivalent to 112 g/week, as 1 unit is equivalent to 8 g of alcohol) for both men and women in the United Kingdom,^34^ and 14 standard drinks per week (equivalent to 196 g/week, because 1 standard drink is equivalent to 14 g of alcohol) for men and 7 standard drinks week (equivalent to 98 g/week) for women in the United States.^35^ The range of maximum recommended alcohol intake is encompassed by the highest intake quartile in EPIC-Norfolk (Table 2); participants in the highest quartile consumed ≥88.78 g/week or 11.09 units/week. The results suggest that alcohol intake within the recommended range, in either the United States or United Kingdom, would be associated with a reduced chance of undergoing cataract surgery.

The mechanism via which alcoholic beverages may protect against cataract development is not clear. Although the fact that some degree of association was present for all types of alcohol beverage suggests that alcohol itself is mediating any potential effect, our observation of strongest associations among wine drinkers, and especially red wine drinkers, also suggests that other components of alcoholic beverages may be contributing. Age-related cataract may result, in part, from oxidative stress to lens proteins.^36^ Dietary intake of antioxidants in alcoholic beverages has been shown to increase plasma antioxidant activity, and this has been hypothesized to reduce cataract formation.^37^ Polyphenols are micronutrients that have antioxidant properties and are present in varying degrees in alcoholic drinks but particularly in wine.^38^ Resveratrol is a natural polyphenol that is found in highest concentrations in red wine. It has strong antioxidant properties and has been hypothesized to potentially protect against several age-related ocular diseases, including cataract.^39^ In a rat model of diabetes, resveratrol supplementation of drinking water delayed the progression of diabetic cataract compared with controls.^40^ Conversely, heavy alcohol consumption induces the expression of microsomal enzyme cytochrome CYP2E1 in the liver. Ethanol metabolism by this enzyme leads to the production of reactive oxygen species,^41^ which in turn may lead to aggregation of lens protein, resulting in cataract development.^42^ Another possible mechanism by which alcohol consumption may reduce cataract risk is via altered cholesterol levels. Alcohol intake has been associated with lower levels of low-density lipoprotein cholesterol (LDL-C),^43^ and LDL-C levels are positively associated with cataract risk.^44^ Therefore, an increase in alcohol intake may lower the risk of cataract via reduced LDL-C. Alcohol intake may reduce LDL-C by decreasing the conversion of very-low-density lipoprotein to low-density lipoprotein apolipoprotein B or increase the clearance of low-density lipoprotein apolipoprotein B.^45^^,^^46^ The biochemical mechanisms may explain the U-shaped association we observed between alcohol intake and cataract surgery in UK Biobank.

### Study Strengths and Limitations

Strengths of our study include its longitudinal design with long-term follow-up and the large sample size of 2 cohort studies, which allowed us to examine the different types of alcoholic beverages in subgroup analyses. Limitations of our study include the self-reported nature of alcohol intake in the 2 cohorts. Participants may underreport or may not accurately recall the amount of alcohol they have consumed or consume combinations of different alcoholic beverage types. However, the misclassification bias is most likely to be nondifferential because information on alcohol consumption was obtained before cataract surgery and thus may bias the effect estimates toward the null. Given our study outcome, cataract is a slowly developing process, and we cannot exclude the possibility that cataract development preceded our exposure assessment of alcohol intake. However, cataract surgery is a hard end point and did follow the exposure measurement timepoint. Although it is not possible to determine direction of causality based on observational studies, it is unlikely that our results are due to cataracts causing reduced alcohol intake, given our longitudinal study design and the significant findings in the dose–response analyses. Because of the chronicity of cataract development, if alcohol has a causal effect, it is likely that this occurs cumulatively over a long period of time. We only ascertained alcohol intake at baseline in both studies and are using this as a surrogate for average intake over a lifetime (i.e., both before and after the baseline assessment). Despite this limitation of ascertainment at only 1 time point, we still identify significant signals that are consistent across 2 studies. Although we adjusted for sociodemographic and lifestyle factors in the multivariable models, it is possible that our imperfectly measured confounders are not fully accounted for and that there are unmeasured confounders we could not account for. Thus, residual confounding may explain our observational associations. However, the clear dose response we have observed may reduce this possibility. Further analysis suggests there is no evidence of multicollinearity between the independent variables. Although we diagnosed cataract by linking to Hospital Episode Statistics data, there was lack of information on the different types of cataract. Therefore, we were unable to examine the associations of alcohol consumption on the various cataract subtypes. Furthermore, as already discussed, cataract surgery is an imperfect surrogate for the development of visually significant cataract.

In conclusion, long-term follow-up data from 2 large longitudinal observational UK cohorts suggest that low to moderate consumption of alcohol may reduce the likelihood of incident cataract requiring surgery. The protective association was particularly pronounced for consumption of polyphenol-rich wine.

## Supplementary Data

Table S6

Application
