# Supplementary material for: Alcohol Consumption and Incident Cataract Surgery in Two Large UK Cohorts
Source: Ophthalmology. 2021 Jun;128(6):837–47. doi: 10.1016/j.ophtha.2021.02.007 (PMC8162662; doi:10.1016/j.ophtha.2021.02.007)
Supplement: Table S1 [file mmc1.pdf]

Table S1. Assessment details for alcohol intake in EPIC-Norfolk and UK Biobank

Variable	Assessment details
**EPIC-Norfolk**	
Total alcohol intake	Uses food tables and standard portion sizes to determine grams of alcohol based on the frequency of the various individual alcoholic drinks.
Wine (glass)	Determined by response to "How often, on average, you have drunk the specified amount of drinks during the past year?" (options: 6+ per day; 4-5 per day; 2-3 per day; once a day; 5-6 per week; 2-4 per week; once a week; 1-3 per month; never or less than once/month).
Beer, lager or cider (half pint)	Determined by response to "How often, on average, you have drunk the specified amount of drinks during the past year?" (options: 6+ per day; 4-5 per day; 2-3 per day; once a day; 5-6 per week; 2-4 per week; once a week; 1-3 per month; never or less than once/month).
Spirits, e.g. Gin, brandy, whisky, vodka (single)	Determined by response to "How often, on average, you have drunk the specified amount of drinks during the past year?" (options: 6+ per day; 4-5 per day; 2-3 per day; once a day; 5-6 per week; 2-4 per week; once a week; 1-3 per month; never or less than once/month).
**UK Biobank**	
Total alcohol intake	Determined by response to "About how often do you drink alcohol?" (options: daily or almost daily, 3 or 4 times a week, once or twice weekly; 1 to 3 times monthly; special occasions only; never).
Average weekly red wine intake	Determined by response to "In an average week, how many glasses of red wine would you drink? (There are six glasses in an average bottle)"
Average weekly champagne plus white wine intake	Determined by response to "In an average week, how many glasses of white wine or champagne would you drink? (There are six glasses in an average bottle)"
Average weekly beer plus cider intake	Determined by response to "In an average week, how many pints of beer or cider would you drink? (Include bitter, lager, stout, ale, Guinness)"
Average weekly spirits intake	Determined by response to "In an average week, how many measures of spirits or liqueurs would you drink? (there are 25 standard measures in a normal sized bottle; spirits include drinks such as whisky, gin, rum, vodka, brandy)"
