# Supplementary material for: Alcohol Consumption and Incident Cataract Surgery in Two Large UK Cohorts
Source: Ophthalmology. 2021 Jun;128(6):837–47. doi: 10.1016/j.ophtha.2021.02.007 (PMC8162662; doi:10.1016/j.ophtha.2021.02.007)
Supplement: Table S2 [file mmc2.pdf]

Table S2. Comparison of baseline characteristics of EPIC-Norfolk participants included and excluded from the study

	EPIC-Norfolk		
	Included	Excluded	*P*-value
Sample size	23,162	2,477	
Age (years), mean (SD)	58.8 (9.2)	63.7 (9.5)	<0.001
Sex, n (%)			<0.001
Men	10,575 (45.7)	1,032 (41.7)	
Women	12,587 (54.3)	1,445 (58.3)	
Ethnicity, n (%)			0.34
White	23,083 (99.7)	2,360 (99.5)	
Non-white	79 (0.3)	11 (0.5)	
Townsend deprivation index, mean (SD)	-2.1 (2.1)	-1.8 (2.3)	<0.001
Body mass index (kg/m^2), mean (SD)	26.3 (3.9)	26.8 (4.2)	<0.001
Smoking status, n (%)			0.001
Never smoked	10,713 (46.3)	961 (42.6)	
Ever smoked	12,449 (53.7)	1,296 (57.4)	
Diabetes status, n (%)			<0.001
No	22,473 (97.0)	2,311 (93.3)	
Yes	689 (3.0)	166 (6.7)	
Alcohol status, n (%)			<0.001
Non-drinker or former drinker	4,516 (19.5)	434 (27.3)	
Current drinker	18,646 (80.5)	1,155 (72.7)	

In EPIC, 106 people had missing ethnicity data, 105 people had missing Townend deprivation index data, 57 people had missing BMI data, 220 people had missing smoking status data and 888 people had missing alcohol status data
