# Supplementary material for: Alcohol Consumption and Incident Cataract Surgery in Two Large UK Cohorts
Source: Ophthalmology. 2021 Jun;128(6):837–47. doi: 10.1016/j.ophtha.2021.02.007 (PMC8162662; doi:10.1016/j.ophtha.2021.02.007)
Supplement: Table S3 [file mmc3.pdf]

Table S3. Comparison of baseline characteristics of UK Biobank participants included and excluded from the study

	UK Biobank		
	Included	Excluded	*P*-value
Sample size	469,387	33,117	
Age (years), mean (SD)	56.3 (8.1)	60.4 (7.4)	<0.001
Sex, n (%)			0.78
Men	214,046 (45.6)	15,076 (45.5)	
Women	255,341 (54.4)	18,041 (54.5)	
Ethnicity, n (%)			<0.001
White	445,610 (94.9)	29,861 (90.2)	
Non-white	23,777 (5.1)	3,256 (9.8)	
Townsend deprivation index, mean (SD)	-1.3 (3.1)	-0.7 (3.3)	<0.001
Body mass index (kg/m^2), mean (SD)	27.4 (4.8)	27.9 (5.0)	<0.001
Smoking status, n (%)			<0.001
Never smoked	258,118 (55.0)	15,404 (51.1)	
Ever smoked	211,269 (45.0)	14,765 (48.9)	
Diabetes status, n (%)			<0.001
No	446,241 (95.1)	29,591 (89.3)	
Yes	23,146 (4.9)	3,527 (10.7)	
Alcohol status, n (%)			<0.001
Non-drinker or former drinker	37,127 (7.9)	3,362 (10.7)	
Current drinker	432,260 (92.1)	28,102 (89.3)	

In UK Biobank, 623 people had missing Townsend deprivation index data, 3,105 people had missing BMI data, 2,948 people had missing smoking status data and 1,653 people had missing alcohol status data.
