# Supplementary material for: Alcohol Consumption and Incident Cataract Surgery in Two Large UK Cohorts
Source: Ophthalmology. 2021 Jun;128(6):837–47. doi: 10.1016/j.ophtha.2021.02.007 (PMC8162662; doi:10.1016/j.ophtha.2021.02.007)
Supplement: Table S4 [file mmc4.pdf]

Table S4. Multivariable dose-response associations with additional adjustment for physical activity

	Multivariable model			
	Number of incident cases	Number at risk	Hazard ratio (95% CI)	*P*-value
**Alcohol consumers in EPIC-Norfolk (n = 18,645)**				
**By quartiles of total weekly alcohol intake**				
Lowest intake (≤14.10 g/ ≤ 1.76 units)	998	4,590	Ref	
Quartile 2 (14.23-43.70 g/ 1.77-5.46 units)	899	4,654	0.92 (0.84-1.01)	0.07
Quartile 3 (43.83-88.53 g/ 5.47-11.07 units)	826	4,644	0.86 (0.79-0.95)	**0.002**
Highest intake (≥ 88.78 g/ ≥ 11.09 units)	732	4,757	0.82 (0.74-0.90)	**<0.001**
P for trend				**<0.001**
**Alcohol consumers in UK Biobank (n = 351,904)**				
**By frequency of alcohol consumption**				
1-3 times or less per month	3,407	82,574	Ref	
1-2 times per week	3,340	97,972	0.92 (0.88-0.97)	**0.001**
3-4 times per week	3,092	90,967	0.94 (0.89-0.99)	**0.012**
Daily or almost daily	3,367	80,391	0.98 (0.94-1.03)	0.51
P for trend				0.75

Multivariable models were adjusted for age, sex, ethnicity, Townsend deprivation index, body mass index, smoking status, diabetes status and physical activity.

Bold values denote statistical significance at the $P<0.05$ level.

Alcohol consumption was quantified by absolute intake in EPIC-Norfolk (presented in both grams and units per week) and by frequency of intake in UK Biobank.

One unit of alcohol (8 grams) is equivalent to one glass of wine, half a pint of beer, lager or cider, or one single measure of spirits.
