# Supplementary material for: Alcohol Consumption and Incident Cataract Surgery in Two Large UK Cohorts
Source: Ophthalmology. 2021 Jun;128(6):837–47. doi: 10.1016/j.ophtha.2021.02.007 (PMC8162662; doi:10.1016/j.ophtha.2021.02.007)
Supplement: Table S5 [file mmc5.pdf]

Table S5. Hazard ratio of incident cataract surgery across different alcohol beverages among drinkers compared to non-drinkers in EPIC-Norfolk, following additional adjustment for physical activity.

	Multivariable model			
Amount of alcohol intake (g)	Number of incident cases	Number at risk	Hazard ratio (95% CI)	*P*-value
Non-drinkers	1,118	4,516	Ref	
Wine drinkers				
First tertile	1,626	7,901	0.98 (0.91-1.06)	0.70
Second tertile	688	4,169	0.82 (0.74-0.90)	**<0.001**
Third tertile	547	3,721	0.77 (0.70-0.86)	**<0.001**
P for trend				**<0.001**
Non-drinkers	1,118	4,516	Ref	
Beer drinkers				
First tertile	985	5,532	0.92 (0.84-1.01)	0.09
Second tertile	392	2,523	0.88 (0.78-0.99)	**0.045**
Third tertile	360	2,567	0.92 (0.80-1.05)	0.21
P for trend				0.09
Non-drinkers	1,118	4,516	Ref	
Spirits drinkers				
First tertile	642	3,591	0.94 (0.85-1.04)	0.23
Second tertile	815	4,523	0.92 (0.83-1.01)	0.07
Third tertile	356	1,842	0.86 (0.76-0.98)	**0.021**
P for trend				**0.012**

Multivariable models were adjusted for age, sex, ethnicity, Townsend deprivation index, body mass index, smoking status, diabetes status and physical activity.

Bold values denote statistical significance at the *P*<0.05 level.
