# Supplementary material for: Alcohol Consumption and Incident Cataract Surgery in Two Large UK Cohorts
Source: Ophthalmology. 2021 Jun;128(6):837–47. doi: 10.1016/j.ophtha.2021.02.007 (PMC8162662; doi:10.1016/j.ophtha.2021.02.007)
Supplement: Table S6 [file mmc6.pdf]

Table S6. Hazard ratio of incident cataract surgery across the different alcohol beverages among drinkers compared to non-drinkers in the UK Biobank, following additional adjustment for physical activity.

| | Multivariable model | | | |
|---|---|---|---|---|
| Number of drinks per week | Number of incident cases | Number at risk | Hazard ratio (95% CI) | *P*-value |
| Non-drinkers | 1,446 | 27,886 | Ref | |
| Red wine drinkers | | | | |
| 1 to 2 glasses/week | 1,965 | 55,754 | 0.85 (0.80-0.92) | **<0.001** |
| 3 to 4 glasses/week | 1,576 | 43,277 | 0.89 (0.82-0.95) | **0.001** |
| ≥ 5 glasses/week | 2,971 | 83,844 | 0.88 (0.82-0.94) | **<0.001** |
| *P* for trend | | | | **0.021** |
| Non-drinkers | 1,446 | 27,886 | Ref | |
| White wine and champagne drinkers | | | | |
| 1 to 2 glasses/week | 2,271 | 63,804 | 0.85 (0.79-0.91) | **<0.001** |
| 3 to 4 glasses/week | 1,234 | 35,194 | 0.87 (0.80-0.94) | **0.001** |
| ≥ 5 glasses/week | 1,751 | 51,232 | 0.91 (0.85-0.98) | **0.018** |
| *P* for trend | | | | 0.28 |
| Non-drinkers | 1,446 | 27,886 | Ref | |
| Beer and cider drinkers | | | | |
| 1 to 2 pints/week | 1,941 | 61,280 | 0.85 (0.79-0.92) | **<0.001** |
| 3 to 4 pints/week | 933 | 28,547 | 0.92 (0.84-1.01) | 0.07 |
| ≥ 5 pints/week | 2,035 | 55,575 | 1.05 (0.96-1.14) | 0.27 |
| *P* for trend | | | | **0.002** |
| Non-drinkers | 1,446 | 27,886 | Ref | |
| Spirits drinkers | | | | |
| 1 to 2 measures/week | 1,929 | 52,192 | 0.87 (0.81-0.93) | **<0.001** |
| 3 to 4 measures/week | 841 | 20,145 | 0.90 (0.82-0.99) | **0.023** |
| ≥ 5 measures/week | 1,365 | 28,637 | 0.98 (0.90-1.06) | 0.62 |
| *P* for trend | | | | 0.69 |

Multivariable models were adjusted for age, sex, ethnicity, Townsend deprivation index, body mass index, smoking status, diabetes status and physical activity.
Bold values denote statistical significance at the *P*<0.05 level.
