# Supplementary material for: Alcohol Consumption and Incident Cataract Surgery in Two Large UK Cohorts
Source: Ophthalmology. 2021 Jun;128(6):837–47. doi: 10.1016/j.ophtha.2021.02.007 (PMC8162662; doi:10.1016/j.ophtha.2021.02.007)
Supplement: Application [file mmc7.docx]

**UK Biobank Eye and Vision Consortium membership**

UK Biobank Eye & Vision Consortium: The UK Biobank Eye & Vision Consortium members are Dr Graeme Black, PhD, The University of Manchester; Panagiotis Sergouniotis, PhD, The University of Manchester; Thomas Littlejohns, PhD, University of Oxford; Naomi Allen, PhD, University of Oxford; Dr Alexander Doney, University of Dundee; Eleni Beli, PhD, Queen’s University Belfast; Denize Atan, PhD, University of Bristol; Tariq Aslam, PhD, Manchester University; Sarah A. Barman, PhD, Kingston University; Jenny H. Barrett, PhD, University of Leeds; Paul Bishop, PhD, Manchester University; Catey Bunce, DSc, King’s College London; Roxana O. Carare, PhD, University of Southampton; Usha Chakravarthy, FRCOphth, Queens University Belfast; Michelle Chan, FRCOphth, NIHR Biomedical Research Centre, Moorfields, London; Sharon Y.L. Chua, PhD, NIHR Biomedical Research Centre, Moorfields, London; David P. Crabb, PhD, City University, London; Alexander Day, FRCOphth, PhD, NIHR Biomedical Research Centre, Moorfields, London; Parul Desai, FRCOphth, PhD, NIHR Biomedical Research Centre, Moorfields, London; Bal Dhillon, FRCOphth, University of Edinburgh; Andrew D. Dick, FMedSci, FRCOphth, University of Bristol; Cathy Egan, FRCOphth, NIHR Biomedical Research Centre, Moorfields, London; Sarah Ennis, PhD, University of Southampton; Paul J Foster, FRCS(Ed), PhD, UCL Institute of Ophthalmology, London; Marcus Fruttiger, PhD, UCL Institute of Ophthalmology, London; John E.J. Gallacher, MSc, PhD, University of Oxford; David F. Garway-Heath MD(Res), FRCOphth, NIHR Biomedical Research Centre, Moorfields, London; Jane Gibson, PhD, University of Southampton; Dan Gore, FRCOphth, NIHR Biomedical Research Centre,Moorfields, London; Jeremy A. Guggenheim, PhD, Cardiff University; Chris J. Hammond, FRCOphth, King's College London; Alison Hardcastle, PhD, UCL Institute of Ophthalmology, London; Simon P. Harding, MD, University of Liverpool; Ruth E. Hogg, PhD, Queens University Belfast; Pirro Hysi, PhD, King's College London; Pearse A. Keane,MD, NIHR Biomedical Research Centre, Moorfields, London; Sir Peng T. Khaw, PhD, NIHR Biomedical Research Centre, Moorfields, London; Anthony P. Khawaja, DPhil, NIHR Biomedical Research Centre, Moorfields, London; Gerassimos Lascaratos, PhD, King's College Hospital NHS Foundation Trust; Andrew J. Lotery, MD, University of Southampton; Tom Macgillivray, PhD, University of Edinburgh; Sarah Mackie, PhD, University of Leeds; Bernadette McGuinness, PhD, Queen’s University Belfast; Gareth J. McKay, PhD, Queen's University Belfast; Martin McKibbin, FRCOphth, Leeds Teaching Hospitals NHS Trust; Tony Moore, FRCOphth, University of California San Francisco; James E. Morgan, DPhil, Cardiff University; Zaynah A. Muthy, BSc, NIHR Biomedical Research Centre, Moorfields, London; Eoin O’Sullivan, MD, King's College Hospital NHS Foundation Trust, Chris G. Owen, PhD, University of London, Praveen Patel, MD(Res), FRCOphth; NIHR Biomedical Research Centre, Moorfields, London; Euan Paterson, BSc, Queens University Belfast; Tunde Peto, PhD, Queen's University Belfast; Axel Petzold, PhD, University College London; Jugnoo S. Rahi, FRCOphth, PhD, Great Ormond Street Hospital & UCL Institute of Child Health, London; Alicja R. Rudnikca, PhD, St George’s University of London; Jay Self, PhD, University of Southampton; Sobha Sivaprasad, FRCOphth, NIHR Biomedical Research Centre, Moorfields, London; David Steel, FRCOphth, Newcastle University; Irene Stratton, MSc, Gloucestershire Hospitals NHS Foundation Trust; Nicholas Strouthidis, PhD, NIHR Biomedical Research Centre, Moorfields, London; Cathie Sudlow, DPhil, University of Edinburgh; Dhanes Thomas, FRCOphth, NIHR Biomedical Research Centre, London; Emanuele Trucco, PhD, University of Dundee; Adnan Tufail, FRCOphth, NIHR Biomedical Research Centre, Moorfields, London; Veronique Vitart, PhD, University of Edinburgh; Stephen A. Vernon, DM, Nottingham University Hospitals NHS Trust; Ananth C. Viswanathan, PhD, FRCOphth, NIHR Biomedical Research Centre, Moorfields, London; Cathy Williams, PhD, University of Bristol; Katie Williams, PhD, King's College London; Jayne V. Woodside, MRCOphth, PhD; Queen's University Belfast, Max M. Yates, PhD, University of East Anglia; Nikolas Pontikos, PhD, University College London and Yalin Zheng, PhD, University of Liverpool.
